# A systematic review of the burden of pertussis in South Korea

**DOI:** 10.1080/21645515.2020.1844505

**Published:** 2021-01-07

**Authors:** Bruce A. Mungall, Hyungwoo Kim, Kyu-Bin Oh

**Affiliations:** a Medical Affairs, GSK, Yongsan-gu, Seoul, Republic of Korea; b Medical Affairs, GSK, Singapore

**Keywords:** Pertussis, Korea, review, epidemiology, immunization, vaccine, seroprevalence, seropositivity

## Abstract

This review summarizes the published data on epidemiology and burden of pertussis in South Korea as these may be under-categorized. A systematic literature review of PubMed, SCOPUS, EMBASE and KMBASE was performed to identify published literature in South Korea since 2000. Pertussis detection rates among 19 eligible studies range from 0.7% to 100% across different age groups, detection methods and study settings. Highest rates are observed in infants, while adolescents and adults with pertussis infection may suffer from persistent coughing. Vaccination uptake of pertussis booster dose among adolescents and adults remains low while seropositivity (detection of anti-pertussis immunoglobulin G), is high among adults. This review reveals a high burden of vaccine-preventable pertussis in South Korea. Besides primary childhood vaccination, strategies like maternal immunization and decennial revaccination of adults should be considered. Active testing, reporting and better utilization of vaccine registries may provide insights for decision-makers nationwide.

## GA

**Figure:**
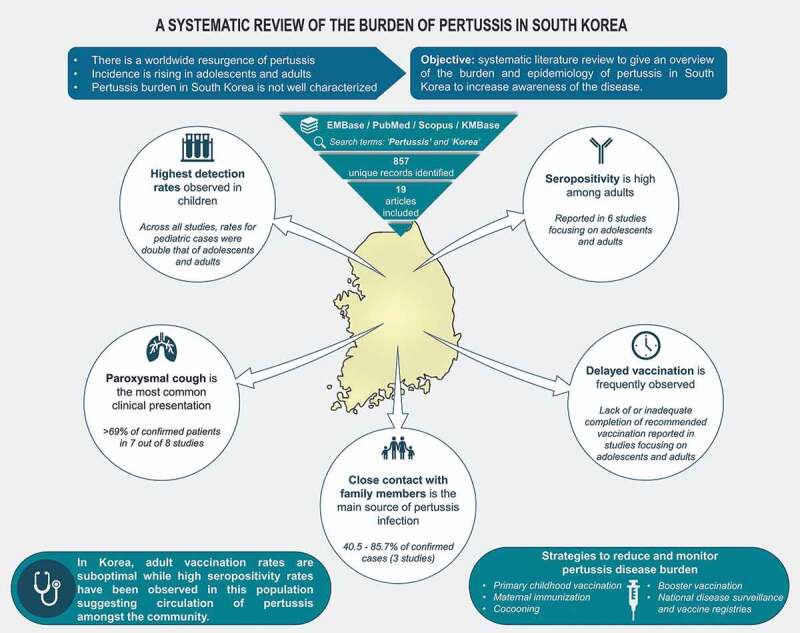

## Introduction

Pertussis, caused by the bacterium *Bordetella pertussis* (*B. pertussis*),^1^ is a highly contagious, vaccine-preventable respiratory disease with a reproductive number (R0) of 15–17 which causes significant childhood morbidity worldwide.^2^ Immunization against pertussis has been in use for several decades.^3^ Despite the successful implementation of primary childhood immunization programs, published evidence indicates a global resurgence of pertussis,^4^ with a rising incidence in adolescents and adults.^5^ This could be due to the fact that immunity to pertussis developed from either natural infection or vaccination is not sustained throughout the lifespan of the individual.^6^ Even if an individual was adequately vaccinated with the diphtheria, tetanus, acellular pertussis (DTaP) vaccine during childhood, additional vaccinations for pertussis during adolescence and adulthood are needed, the lack of which would make the individual susceptible to pertussis infection.^6^ These adolescents and adults further act as a significant infectious source of pertussis in infants, specifically those who are too young to be vaccinated or are incompletely immunized.^7,8^

Considering this situation, the World Health Organization provided an update of its recommendations on pertussis surveillance.^9^ Data on the changing epidemiology of pertussis are needed to ensure that pertussis control policies in all countries are still pertinent. Despite the disease being notifiable in many countries, data on pertussis disease trends are only available from high-income countries and data is generally lacking from low- and middle-income countries.^10^ In particular, the epidemiology of pertussis disease in the Asia-Pacific region has received little attention.^11^

In South Korea, pertussis is a notifiable disease since 1954 when the whole-cell pertussis vaccine was first recommended.^12^ After this in 1989, the DTaP vaccine was introduced into the Korean National Immunization Program (NIP), and currently, DTaP vaccine is given with 3 + 1 schedule and as a pre-school booster.^13^ Moreover, a tetanus toxoid, reduced diphtheria and acellular pertussis (Tdap) vaccine have been recently included (2012) in the Korean NIP and is recommended as the first booster immunization for individuals at 11–12 years (y) of age.^14^ A single dose of Tdap is also recommended for adults over 18 y followed by decennial booster dose of Td.^15^ Vaccination coverage rates in 2020 are estimated to be at 98.9%,^15^ 70% and 5.6% in children receiving the first dose of the DTaP vaccine, and expectant mothers and adults >50 y of age receiving the Tdap vaccine, respectively (source: personal communication). While the low incidence of diphtheria and tetanus has been maintained among the Korean population, the incidence of pertussis has rapidly increased in South Korea since 2009.^16,17^ Despite this situation in South Korea, the burden of pertussis disease does not appear to be well characterized.

To our knowledge, there are a limited number of published studies on pertussis disease burden and epidemiology in South Korea and there has been no attempt to systematically review and collate data from published studies. We conducted a systematic literature review to consolidate existing data on the epidemiology and burden of disease of pertussis in South Korea with the objective to provide a national overview of the burden of disease and highlighting evidence gaps which could help improve awareness about pertussis disease in South Korea.

## Methods

**Figure 1.**
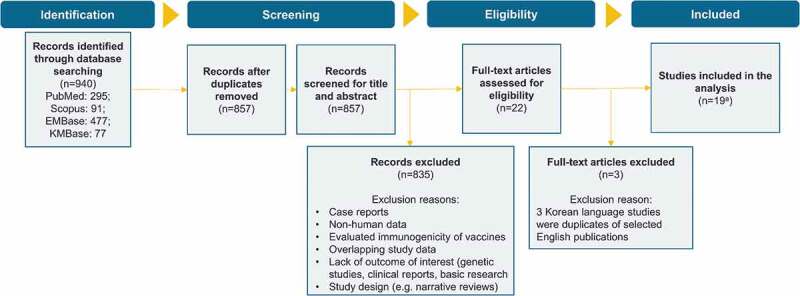
PRISMA flow diagram

^a^12 articles in English and 7 articles in Korean language

This review was conducted according to guidelines in the Cochrane Handbook for Systematic Reviews of Interventions,^18^ and Preferred Reporting Items for Systematic Literature Reviews and Meta-Analyses (PRISMA)^19^ to obtain relevant information using a reproducible, robust and transparent methodology. In-line with these guidelines, a review protocol detailing the review method was developed. This protocol was registered on the International Prospective Register of Systematic Reviews (PROSPERO: CRD42020165408, review in progress). The PRISMA flow chart is represented in Figure 1 and details on search strategy and eligibility criteria can be found in Supplementary Table 1.

Studies were reviewed to determine pertussis detection rates, stratified by study design, diagnostic technique, population characteristics, clinical symptoms, source of infection and vaccination history.

### Risk of bias analysis

Studies included in this review had minimum bias risk. Across all the studies, the sample frame was considered appropriate to address the target population and the study participants were sampled in an appropriate way with an adequate sample size. With the exception of Kim et al.^20^ and Lee et al.,^21^ study subjects and the setting were described in detail. Among the 19 studies, analysis was conducted with sufficient coverage of the identified sample. Valid methods were used for the identification of the condition with the exception of the study by Lee et al.^22^ in which only immunoglobulin G (IgG) titer was measured. The condition was measured in a standard, reliable way for all participants with the exception of the Choe et al.^23^ study in which it was not clear how this was done prior to the year 2001 (note that data prior to 2001 from this study were not included). Appropriate statistical analysis within each publication was done and the response rate was adequate or managed appropriately if the response rate was low with the exception of Son et al.^24^ (Supplementary Table 2).

## Results

### Study characteristics

**Figure 2.**
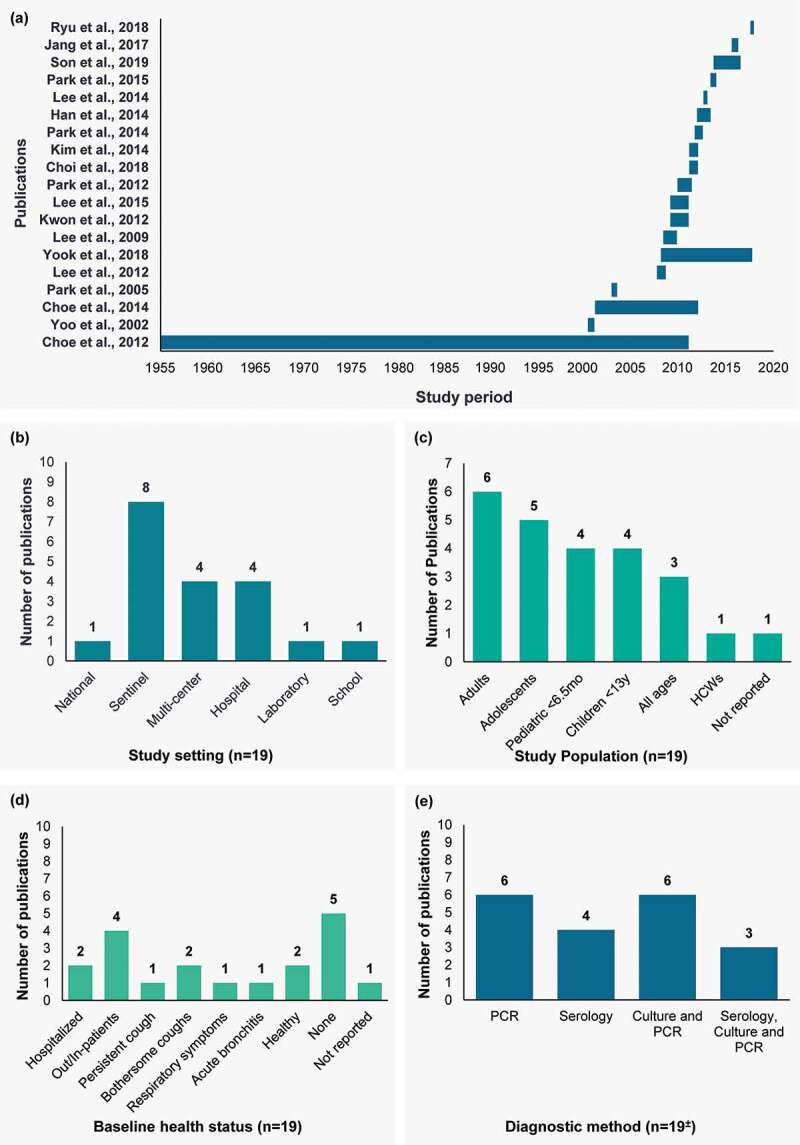
Study characteristics. (a) Study period, (b) study setting, (c) study population, (d) baseline health status, and (e) diagnostic method

HCW, healthcare worker; m, months; PCR, polymerase chain reaction.^a^The total number of studies depicted in the figure is 24 as five studies reported information for more than one group.^22,24–27^

The studies included in this review report data on pertussis epidemiology and burden in South Korea from 1955 until 2017. There were four studies which reported data for periods between the years 2000 and 2009 (10 y). Nine studies reported data for periods between the years 2009 and 2013 (5 y) and 3 studies presented data for periods between the years 2013 and 2017 (5 y). Of the remaining studies, one study evaluated pertussis incidence between the years 1955–2011 (57 y) and presented data on cases between the years 1955 and 2000, limited data from 2001 to 2009 and detailed data from 2010 to 2011. Another study presented data for the years 2001–2012 (12 y) and one study evaluated the years 2008–2017 (10 y) (Figure 2a; Table 1).

**Table 1.** Proportion of cases with positive detection of pertussis (*n* = 19)

Author (year)	Time period	Type of study	Location	Age population	Health status	Study population	Method utilized	Lab confirmed	PCR	Culture	Seropositivity
Choe et al. (2012)^23^	2010–2011	National passive surveillance	Primary care clinics and hospitals	All ages	Clinically suspected case	124 suspected cases <3 months (*n* = 42) 3–5 months (*n* = 18) 6–11 months (*n* = 6) 1–4 y (*n* = 12) 5– 9 y (*n* = 7) 10–1 4 y (*n* = 3) >5 y (*n* = 36)	Culture and PCR	Overall (*n* = 95, 76.6%) <3 months (*n* = 31, 73.8%) 3–5 months (*n* = 15, 83.8%) 6–11 months (*n* = 5, 83.3%) 1–4 y (*n* = 10, 83.3%) 5–9 y (*n* = 5, 71.4%) 10–14 y (*n* = 2, 66.7%) >15 y (*n* = 27, 75%)			
Choe et al. (2014)^28^	2001–2012; 2012 (Mar–Jul)	National passive + active sentinel surveillance in school during outbreak	Primary care clinics, hospitals and schools	All ages	Clinically suspected case	2001–2012: 416 suspected cases 2012: 496 suspected cases	PCR		13.0%		
Choi et al. (2018)^29^	2011	Cross-sectional	A hospital	Adults	Healthy HCW	398 subjects	Serology				66.3% IgG <5 IU/mL 30.4% IgG 5–40 IU/mL 2.5% IgG 40–100 IU/mL 0.8% IgG >100 IU/mL Overall 33.7%
Han et al. (2014)^30^	2011 (Oct)–2013 (Apr)	Prospective observational	A hospital	Infants under 6 months	Hospitalized patients with lower respiratory tract infection	79 suspected patients	Culture and PCR	16.5%	16.5%	0.0%	
Jang et al. (2017)^25^	2015 (Jun)–2016 (Mar)	Prospective observational	A hospital	Children and adolescents under the age of 18	Clinically suspected cases in inpatients and outpatients	50 suspected patients	Serology, culture and PCR		4.0%	0.0%	74.0%
Kim et al. (2014)^20^	2011–2012 (2009 follow-up study)	Laboratory					PCR				
Kwon et al. (2012)^31^	2009–2011	Prospective observational	Multicenter	Children	Clinically suspected cases in inpatients and outpatients	65 infants (^a^72 family members and care-givers)	Serology, Culture and PCR	32.3%/52.8%	32.3%/52.8%	13.8%/-	–/100.0% (31/31)
Lee et al. (2009)^21^	2008 (Mar)–2009 (Sep)	Retrospective observational	A hospital	Children and adolescents under the age of 18	Clinically suspected cases in inpatients and outpatients	118 pediatric	PCR	8.5%	8.5%		
Lee et al. (2012)^32^	2007 (Jul)–2008 (Jul)	Prospective observational	Multicenter	All ages	Healthy children, adolescents and adults	≤10 y (*n* = 1,005) 11–20 y (*n* = 100) 21–30 y (*n* = 100) 31–40 y (*n* = 100) 41–50 y (*n* = 100) 51–60 y (*n* = 100) ≥61 y (*n* = 100)	Serology				68.2% (2 months–65 y) 76.5% (<11 y)
Lee et al. (2015)^27^	2009–2011 (Dec)	Prospective observational	Two hospitals	Adolescent (aged 11–20 y), adults (>20 y)	Patients with persistent cough of 1–8 weeks	310 Suspected cases	Serology, culture and PCR	24.5%	3.2%	1.0%	21.3%
Lee et al. (2014)^22^	2012 (Jun–Dec)	Retrospective observational	Two hospitals	Adolescent, adults	Healthy adolescents and adults	11–20 y (*n* = 198) 21–30 y (*n* = 211) 31–40y (*n* = 213) 41–50 y (*n* = 198) 51–60 y (*n* = 191) ≥61 y (*n* = 181)	Serology				Average: 41.4% >51 y: 46.5% <51 y: 39.1%
Park et al. (2012)^33^	2009 (Sep)–2011 (Apr)	Prospective observational	Primary care clinics and hospitals	Adults	Outpatients (≥18 y) with bothersome cough	^b^607 suspected cases/934 total subjects	PCR	0.7%/0.5%			
Park et al. (2014)^26^	2011 (Jul)–2012 (Jun)	Prospective observational	Primary care clinics and hospitals	Adolescent, adults	Clinically suspected cases in outpatients (≥11 y)	490 auspected cases	Culture and PCR	6.9%	6.9%	2.0%	
Park et al. (2015)^34^	2013 (Mar–Jun; Oct–Nov)	Prospective observational	Primary care clinics and hospitals	Adults	Patients with acute bronchitis	435 acute bronchitis	PCR	0.7%	0.7%		
Park et al. (2005)^35^	2002 (Sep)–2003 (May)	Prospective observational	Two hospitals	Adults	Patients with persistent cough of 1–12 weeks	102 suspected cases	Culture and PCR	2.9%	2.9%	0.0%	
Ryu et al. (2018)^36^	2017 (Jun–Nov)	Outbreak investigation	An elementary school	Children	Clinically suspected cases	9 suspected cases	Culture and PCR	100.0%	88.9%	11.1%	
Son et al. (2019)^24^	2013 (Jul)–2016 (Jun)	Prospective observational	Multinational	Children and adolescents	Children or adolescents who had not received pertussis vaccine within 1 y	139 subjects	Serology				10–11 y: 2.2% 12–18 y: 0.0%
Yoo et al. (2002)^37^	2000 (Mar)–2001	Prospective observational	A hospital	Children	Clinically suspected cases in hospitalized children	49 suspected cases	Culture and PCR	20.4%	20.4%	8.2%	
Yook et al. (2018)^38^	2008 (Jan)–2017 (Sep)	Retrospective observational	A hospital	Children (cases <13 y)	Hospitalized patients with bacterial pneumonia	1,281 bacterial pneumonia	PCR		1.3%		

^a^Only infants were included in the current publication analysis.

^b^Suspected cases are subjects with bothersome cough.

IgG, immunoglobulin G; IU/ml, international units per milliliter; PCR, polymerase chain reaction; y, year(s).

There was a total of 16 active surveillance studies, out of which 4 were conducted in hospitals, 6 were conducted in sentinel sites, 4 were conducted across multi-center locations, one was from a laboratory and one was from a school. There were two passive surveillance studies, out of which one was from a national site and one from a sentinel site. Lastly, one study was a combination of passive and active surveillance from a sentinel site (Figure 2b; Table 1).

Of the 19 publications included in this review, 6, 5, 4, 4 and 3 studies reported data for adults, adolescents, young children (<6.5 months of age), children (<13 y of age) and all ages, respectively. Of the remaining studies, one study reported data for healthcare workers (HCW) and one study did not report the age of the study population. In addition to the 3 studies reporting across all ages, 5 studies reported data across multiple age groups (Figure 2c; Table 1).

Apart from two studies in healthy individuals; data were reported for populations with a varied baseline health status: hospitalized (n = 2), out- and in-patients (n = 4) and presenting with persistent cough (n = 1), bothersome coughs (n = 2), respiratory symptoms (n = 1) and acute bronchitis (n = 1). Suspected cases were reported in 3 studies and no details of morbidity or risk factors were provided in 3 studies (Figure 2d; Table 1).

Diagnostic methods used to detect cases of pertussis were reported in 19 publications. An equal number of studies reported the use of polymerase chain reaction (PCR) methodology alone (n = 6) and the use of both culture and PCR methods (n = 6). Four studies reported the use of serology alone and 3 studies reported the use of serology, culture and PCR methods (Figure 2e; Table 1).

### Detection of pertussis cases

Substantial variation in reporting between studies was apparent due to differences in diagnostic techniques and study populations. Among the 19 studies in this review, 12 studies reported prevalence data in South Korea based on laboratory-confirmed cases of *B. pertussis* with a detection rate ranging from 0.7% to 100% across different age groups, detection methods and study settings. A total of 13 studies utilized the PCR methodology for *B. pertussis* detection and reported a detection rate ranging from 0.7% to 88.9%. Eight studies utilized the culture methodology for *B. pertussis* detection with a detection rate ranging from 0.0% to 13.8% (Table 1).

The age-specific detection rate of *B. pertussis* cases in South Korea was reported in two studies which included age groups ranging from below 3 months of age to greater than 15 y of age.^23,38^ The highest detection rate by laboratory confirmation in patients with suspected pertussis was reported in very young children (>3 months to 4 y of age) followed by adolescents and adults >15 years of age.^23^ In another study, pertussis detection rate (PCR) in patients hospitalized with respiratory symptoms was more than double in adolescents and adults of ages 10 to 20 y (2.7%) compared to children below 10 y (1.2%).^38^ Combining data from all studies, laboratory confirmation rates for pediatric cases were numerically double that of adolescents and adults (35.5% [standard error: ±18.5%] versus 15.8% [standard error: ±6.5%]) although substantial variation was apparent between studies.

While the sensitivity of diagnostic modality varies between the studies included in this review and appears to be dependent on the methodology, technique and sample source (nasopharyngeal, sputum and throat swab), with such a small number of studies it is inherently difficult to determine consistent trends. There is significant variation in the pertussis case detection rates by study population and methodology (single and multiple detection modality) compared to those using individual techniques, i.e. detection rates tend to be higher if a combination of diagnostic techniques were used. Nasopharyngeal aspirates and swabs appear to be associated with higher rates of detection, but again, the small number of studies limits definitive conclusions (Table 1; Supplementary Figure 1).

### Seropositivity

Seropositivity can be an indication of recent or previous pertussis exposure depending on the level of antibodies detected. Most studies reported using a cutoff of anti-pertussis toxin (PT) IgG titer >24 international units per milliliter (IU/mL) as an indication of positivity, representing previous exposure to pertussis. Additionally, a small number of studies also stratified subjects according to a higher anti-PT IgG titer (usually ≥100 IU/mL) as indicative of recent or acute exposure, this latter group can also be confirmed by anti-PT IgA ELISA. Data based on the detection of anti-PT IgG, stratified according to kit manufacturer’s instructions were presented in 6 studies and ranged from 0.0% to 76.5%. Of these, 3 studies were conducted in children and 6 studies were conducted in adolescents and adults. A total of 3 studies reported age-specific seropositivity data. Lee et al.^32^ reported an overall seropositivity rate of 68.2% (age group: 2 months-65 y) with the highest seropositivity rate in children below 11 y of age (76.5%). In another study, seropositivity rate was higher in adults >51 y of age (46.5%) compared to individuals <51 y of age (39.1%).^22^ In contrast, a study in HCWs reported an overall seroprevalence rate of pertussis antibodies of 33.7% with recent, acute infection suspected in 0.8% (Table 1).^29^

### Clinical presentation

Clinical presentations associated with *B. pertussis* were reported in 9 studies included in this review,^21,25,26,29,31,33–36^ of which the majority were conducted in adolescents and adults.^25,26,29,33–35^ Regardless of age, the most common presentations of pertussis were paroxysmal cough followed by whooping cough, post-tussive vomiting and fever. Cough duration was similar in the pediatric (average: 22.2 ± 11.8 days [d]; range: 10–45.8 d) and adolescent and adult populations (average: 16.5 ± 3.7 d; range: 7–30 d)^21,26,31,34^; however, pertussis tended to be a cause of chronic cough in adult patients.^33–35^

The most common clinical presentation in pertussis cases was paroxysmal cough followed by post-tussive vomiting (Supplementary Figure 2). Characteristic “whooping” cough was only present in approximately 25% of the cases. Notably, cough duration prior to diagnosis appeared to be of lower predictive value for pertussis diagnosis in adults versus children, while paroxysmal cough was highly associated with detection, with more than 69% of confirmed patients presenting with this symptom in 7 out of 8 studies (Supplementary Figures 3 and 4).

### Impact of vaccination and pertussis disease burden

The vaccination status of confirmed or suspected pertussis cases was reported in 8 studies.^21,23,27,29,31,33,36,37^ A total of five studies that focused on the pediatric population reported that the majority of the subjects delayed vaccination or were not vaccinated according to the recommended schedules.^21,23,31,36,37^ Choe et al.^23^ reported that 25% of the infants of ages 3–11 months did not receive the diphtheria, tetanus, pertussis (DTP) vaccine as per the schedule and there was no record of immunization in 97.7% of the adolescents and adults. Kwon et al.^31^ reported that 42.9% of the infants <6 months old had not received any DTaP vaccination, while 42.9%, 9.5% and 4.8% of the children received 1, 2 and 3 vaccine doses, respectively. In the same study, vaccinated children had a shorter length of hospitalization (8.8 ± 3.8 d) than unvaccinated children (15.4 ± 6.6 d), respectively. In another study conducted in children (9 of 10 were <3 months old), 70% of the cases were not immunized; notably, though no pertussis-related complications were documented in vaccinated children compared to complications in 6 out of 7 unvaccinated children.^21^ In the study by Yoo et al.,^37^ 80% of the infants (average age 4.6 months) were not immunized. In contrast, Ryu et al.^36^ reported a 100% vaccination completion rate among suspected cases 7–10 y old; importantly vaccination had a positive effect on the severity of the disease.

Three studies focusing on the adult and adolescent population also demonstrated a lack of or inadequate completion of recommended vaccination.^27,29,33^ One study among HCWs in South Korea reported that none of the participants were vaccinated against pertussis since childhood.^29^ In a study of 310 suspected cases of pertussis in adolescents and adults, none of the subjects received booster vaccination against pertussis within the 5 y prior to the study,^27^ whereas in another study, among 607 suspected cases, only 13.2% of the adults were vaccinated (DTaP or DTP).^33^

### Source of infection

A total of 9 studies in this review reported data on source of infection.^21,23,24,26,29,31–33,36^ Out of these, 3 studies reported that close family members were the source of infection of laboratory-confirmed cases ranging from 40.5% to 85.7% of the infants.^21,23,31^ Notably, one study showed that mothers were the source of a large proportion of cases in infants (34.2%).^31^ Four studies reported that proximity to individuals, who were positive for *B. pertussis* infection or had cough symptoms, was responsible for the spread of the disease among children, adolescents and adults in South Korea.^24,26,33,36^

### Discussion

This systematic literature review summarizes the incidence and disease burden of pertussis in South Korea based on data from 19 studies conducted in diverse settings among individuals of all ages. A number of important observations are apparent from this study: first, the included studies reveal gaps about pertussis epidemiology in South Korea. The studies provide data from time-points across the entire 20-y scope of this review, the majority of them from sentinel and multi-center settings suggesting that these data may be extrapolated and generalized, albeit cautiously, to the national level. Studies were also reasonably evenly distributed across all age groups, thus supporting the generalization of results to the general population. While data were also reported for populations with a varied baseline health status, there was limited information available on the existence of underlying conditions and the role these may or may not play in susceptibility to pertussis infection.

Second, this review demonstrates that detection methodologies have not been consistently applied, leading to a substantial variation in pertussis detection rates. Detection rates were robust in studies that utilized multiple methods compared to those studies using only a single technique to detect infection rates. The simultaneous use of all three diagnostic methods (culture, PCR and serology) yielded the highest pertussis confirmation rates. These rates were comparable to detection rates from studies that used both PCR and culture or only serology. Pertussis detection rates varied among studies that utilized single diagnostic methods. When data were stratified by individual diagnostic methods, serologic diagnosis yielded the highest confirmation rates. The most commonly used method to diagnose pertussis was PCR; however, it yielded lower rates of confirmation when used alone compared to serologic diagnosis alone. It is known that the time since disease onset (cough onset) influences the sensitivity of detection techniques with the sensitivity of culture and PCR diagnosis being high early during disease onset, but declining considerably after the first two weeks.^39^ In contrast, serology performed early in the course of disease has low sensitivity and is optimal after the first weeks, suggesting that in several studies, samples were collected later into the disease than would be considered optimal. Importantly, PCR or culture-positive tests suggest acute infection in patients with clinically suspected symptoms while positive results by a single serologic test may not represent acute infections. To confirm acute infection by serologic testing, it is necessary to follow-up with acute and convalescent serum samples. However, paired serologic tests are not routinely conducted in South Korea.^40^ Additionally, serology for pertussis diagnosis in pediatric populations is challenging as results may be difficult to interpret in vaccinated children and are documented to have longer turnaround times compared to other techniques for detection.^41^ To determine pertussis infection, the individual studies utilized the nasopharyngeal aspirate or swab, the sputum or the throat swab as the sample source for laboratory confirmation among the PCR and culture methodologies. The nasopharyngeal aspirate or swab was associated with the highest rates of detection among both diagnostic methodologies (PCR and culture) and are therefore preferred as the source sample for the detection of pertussis. Further studies are required to understand if serologic diagnosis alone (preferably as paired samples) is more efficient than utilizing only PCR or culture diagnostic methods.^42^ This information will be useful in informing recommendations for pertussis testing in South Korea.

Thirdly, despite limited data being available on the age-specific occurrence of pertussis, some insights can be gleaned from this review. In comparing age-specific- pertussis detection rates, the highest rates were reported in infants and young children followed by adolescents and adults. Similarly, our analysis suggests that pertussis detection rates in pediatric cases are double the rates observed in adolescents and adults. Notably, overall detection rate estimates reflect a high pertussis disease burden in South Korea, though disease burden could potentially be underreported in very young infants (below 6 months of age) and among adolescents and adults. This may be due to the use of presumptive antibiotics and the lack of reimbursement for diagnostic testing in South Korea, both of which could contribute to the underestimation of the pertussis disease burden.^34,43^ There was very limited data available for older adults, highlighting the need for specific pertussis epidemiology and disease burden studies.

Fourthly, this review highlights gaps in pertussis vaccination implementation in South Korea. National vaccination data indicate the childhood immunization program is successful in Korea, although this is slightly lower for booster immunization including those for adolescents. Maternal immunization and adult vaccine uptake rates are also suboptimal (source: personal communication). Consistent with this, we found studies focusing on the adolescent and adult populations, including HCWs which reported a lack of, or inadequate completion of recommended vaccines. This could be due to the lack of reporting vaccination for adults. The KCDC vaccination guideline state that vaccination history is to be documented in the registry system, while in reality, the use of this registry is very low.^15^ There is an urgent need to improve awareness and uptake of the existing vaccination registry system made available by the KCDC^15^ which will help identify individuals who need vaccination. On the other hand, if vaccination uptake rates in adults are indeed low, the observation of high seropositivity might also suggest natural boosting due to recent infection with circulating strains of *B. pertussis* in Korea. Regardless, vaccination strategies such as maternal immunization and decennial revaccination are urgently required to reduce natural pertussis infection in adults as they are known to play an important role in pertussis transmission to young children.^21,23,31^ This becomes critical when children are not vaccinated or are too young to be vaccinated, leaving them vulnerable to infection. In this context, while the military tender in Korea has served as a significant intervention in pertussis control (246,000 doses per year; personal communication), it is only for half the adult population and only a one-time vaccination for new recruits (potentially 18–25 y-olds). Seropositivity rates, as an indication of recent or previous pertussis exposure, are high in adults as reported in several studies in this review. According to the KCDC, decreasing tetanus/diphtheria antibody titer with age and sustaining pertussis antibody titer may be suggestive of circulating pertussis in the community.^16^ Consequently, vaccination uptake in this population deserves urgent attention.

Lastly, the clinical presentations associated with *B. pertussis* such as cough followed by paroxysmal cough, whooping cough and post-tussive vomiting are consistent with that reported elsewhere, ^44^ and may also be informative for clinician awareness. Our data indicate that over 80% of the confirmed cases presented with paroxysmal cough, suggesting some predictive value of this clinical observation (Supplementary Figure 2). However, much lower rates of post-tussive vomiting and characteristic “whooping” cough were typically observed, with fever observed in less than 15% of the cases on average. Cough duration was of slightly longer duration in pediatric cases compared to adolescent and adult populations. Pertussis tended to be a cause of chronic cough in adult patients; however, cough duration was determined to be of low predictive value in the detection of pertussis infection, possibly due to bias in the different selection criteria used across the studies.

Strengths of this review include the use of a systematic methodology that helped provide a comprehensive evidence base on the existing burden of pertussis in South Korea. Studies were identified as having minimum risk of bias with comprehensive representation of the general population of South Korea. We acknowledge that there are some limitations to this work. Studies included in this review did not specifically report on the actual incidence of pertussis; however, the case detection rates do provide meaningful insights into the disease profile of the country. Moreover, the type of surveillance (active or passive) was extracted as reported in the original article. Restricting the language of publications could introduce bias; however, the choice of English or Korean was considered appropriate to capture relevant literature for the context of South Korea. We also restricted our search to identify publications from theyear 2000 and after. This was done as published epidemiological data on pertussis more than 20 y old were considered of low relevance to discussions on the current burden of disease. Moreover, in the context of healthcare policy and decision-making, recent epidemiological data are considered relevant.

### Conclusions

It is likely that pertussis disease burden is largely underestimated or underreported in South Korea due to diagnostic challenges and awareness. While there is a need for nationwide surveillance, increasing awareness would result in a higher detection rate through more proactive testing practices. This is supported by the finding that the highest detection rates were among young children in which more often pertussis is considered as a differential diagnosis. There are clear gaps in the surveillance for pertussis epidemiology in South Korea and specific, robust studies would be helpful to estimate the burden of pertussis considering that nasopharyngeal swab is the gold standard for pertussis diagnosis. The NIP has been successful in Korea for infants, children, and adolescents, particularly for pertussis vaccine uptake. However, Tdap booster uptake rates are suboptimal among adults, expectant mothers and HCWs. Considering that circulating community pertussis could be transmitted to infants who are too young to be vaccinated and pertussis among adults itself is a burden on the health-care system and has an impact on daily life, increasing adult Tdap booster dose uptake rates together with maternal immunization would be helpful for pertussis control. To facilitate adult vaccination uptake, the development and implementation of a whole of life vaccination registry would be helpful to increase Tdap vaccination coverage.

## Supplementary Material

Supplemental Material

## Highlights

- In South Korea, pertussis detection rates ranged from 0.7% to 100%.
- Highest rates were observed in young infants followed by adolescents and adults.
- Close contact with family members is the main source of pertussis infection.
- Uptake of pertussis vaccine booster dose among adolescents and adults remained low.
- High IgG seropositivity in adults suggests circulation of community pertussis.
