# Supplementary material for: A systematic review of the burden of pertussis in South Korea
Source: Hum Vaccin Immunother. 2021 Jan 7;17(6):1747–56. doi: 10.1080/21645515.2020.1844505 (PMC8115764; doi:10.1080/21645515.2020.1844505)
Supplement: Supplemental Material [file KHVI_A_1844505_SM6099.docx]

# A systematic review of the burden of pertussis in South Korea

# Supplementary material

## Supplementary Table 1: Search strategy and eligibility criteria

| **Search sources** | **Search strategy** | |
| --- | --- | --- |
| PubMed | (pertussis) AND Korea) AND ("2000/01/01"[Date - Publication]: "3000"[Date - Publication]) | |
| SCOPUS | TITLE-ABS-KEY (pertussis AND korea) AND PUBYEAR > 1999 | |
| EMBASE | pertussis AND korea AND [2000-2020]/py | |
| KMBASE | “Pertussis” AND “Korea”, publication year “2000” – “2020” | |
| **Criteria** | **Inclusion criteria** | **Exclusion criteria** |
| **Population** | - All ages | - None |
| **Intervention/Comparator** | - Not restricted by intervention or comparator | - None |
| **Outcome** | - Number of cases or percentage (overall and by age or baseline heal.th status, if available) - Incidence of pertussis (overall and by age or baseline heal.th status, if available) - Measures of disease burden (seropositivity, clinical. characteristics, vaccination status and source of infection) - Timing and effect measures (e.g. time between symptom onset and testing and relation with diagnostic testing according to technique used, differences in symptoms/outcomes between vaccinated and unvaccinated individuals, etc.) | - All other outcomes |
| **Study design** | - Quantitative studies and qualitative studies | - Meta-analysis - Letter to editor - Newspaper - Editorial. - Comment - Opinion paper - Reviews |
| **Time limit** | - 2000 onwards | - None |
| **Language** | - English, Korean | - All other languages |
| **Geographic scope** | - South Korea | - All other countries |

py, publication year

## Supplementary Table 2: Risk of bias analysis (n=19)

| **Publication** | 1. Was the sample frame appropriate to address the target population? | 2. Were study participants sampled in an appropriate way? | 3. Was the sample size adequate? | 4. Were the study subjects and the setting described in detail? | 5. Was the data analysis conducted with sufficient coverage of the identified sample? | 6. Were val.id methods used for the identification of the condition? | 7. Was the condition measured in a standard, reliable way for all participants? | 8. Was there appropriate statistical. anal.ysis? | 9. Was the response rate adequate, and if not, was the low response rate managed appropriately? |
| --- | --- | --- | --- | --- | --- | --- | --- | --- | --- |
| Choe et al., 2012^1^ | Yes | Yes | Yes | Yes | Yes | Yes | Unclear pre-2001 | Yes | NA |
| Choe et al., 2014^2^ | Yes | Yes | Yes | Yes | Yes | Yes | Yes | NA | NA |
| Choi et al., 2018^3^ | Yes | Yes | Yes | Yes | Yes | Yes | Yes | Yes | Yes |
| Han et al., 2014^4^ | Yes | Yes | Yes | Yes | Yes | Yes | Yes | Yes | NA |
| Jang et al., 2017^5^ | Yes | Yes | Yes | Yes | Yes | Yes | Yes | Yes | NA |
| Kim et al., 2014^6^ | Yes | Yes | Yes | No | Yes | Yes | Yes | Yes | NA |
| Kwon et al., 2012^7^ | Yes | Yes | Yes | Yes | Yes | Yes | Yes | Yes | Yes |
| Lee et al., 2009^8^ | Yes | Yes | Yes | No | Yes | Yes | Yes | Yes | NA |
| Lee et al., 2012^9^ | Yes | Yes | Yes | Yes | Yes | Yes | Yes | Yes | NA |
| Lee et al., 2015^10^ | Yes | Yes | Yes | Yes | Yes | Yes | Yes | Yes | NA |
| Lee et al., 2014^11^ | Yes | Yes | Yes | Yes | Yes | IgG titer only | Yes | Yes | NA |
| Park et al., 2012^12^ | Yes | Yes | Yes | Yes | Yes | Yes | Yes | Yes | Yes |
| Park et al., 2014^13^ | Yes | Yes | Yes | Yes | Yes | Yes | Yes | Yes | Yes |
| Park et al., 2015^14^ | Yes | Yes | Yes | Yes | Yes | Yes | Yes | Yes | Yes |
| Park et al., 2005^15^ | Yes | Yes | Yes | Yes | Yes | Yes | Yes | Yes | Yes |
| Ryu et al., 2018^16^ | Yes | Yes | Yes | Yes | Yes | Yes | Yes | Yes | Yes |
| Son et al., 2019^17^ | Yes | Yes | Yes | Yes | Yes | Yes | Yes | Yes | Unclear |
| Yoo et al., 2002^18^ | Yes | Yes | Yes | Yes | Yes | Yes | Yes | NA | NA |
| Yook et al., 2018^19^ | Yes | Yes | Yes | Yes | Yes | Yes | Yes | Yes | Yes |

NA, not applicable; IgG, immunoglobulin G

##
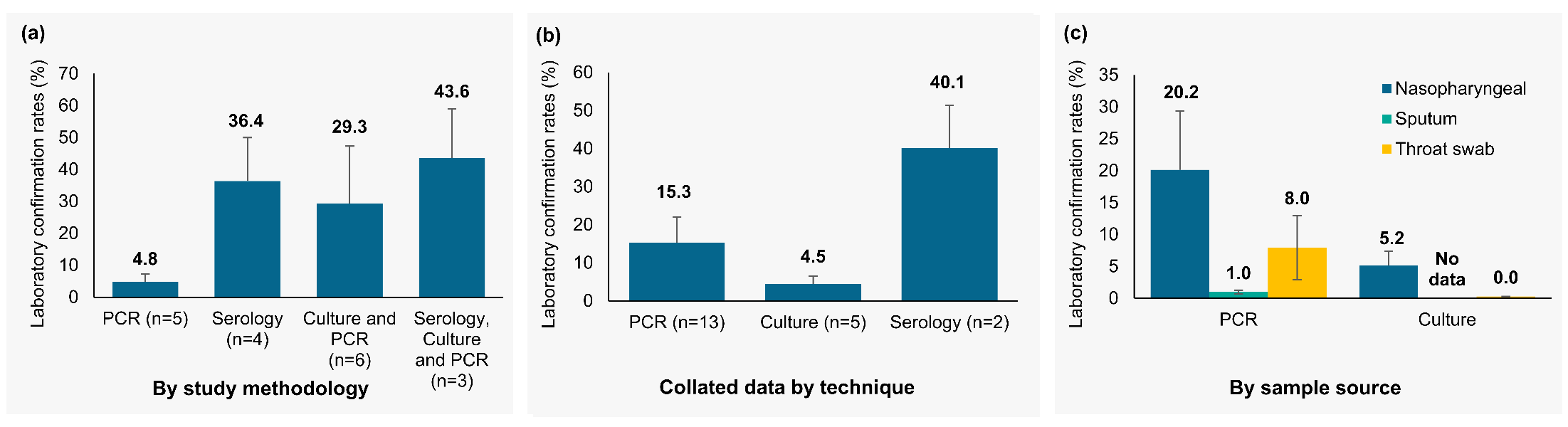
Supplementary Figure 1: Laboratory confirmation rates (a) Study methodology (b) Technique (c) Sample source

PCR, polymerase chain reaction

Error bars: standard error

## Supplementary Figure 2: Presence of clinical characteristics in pertussis cases


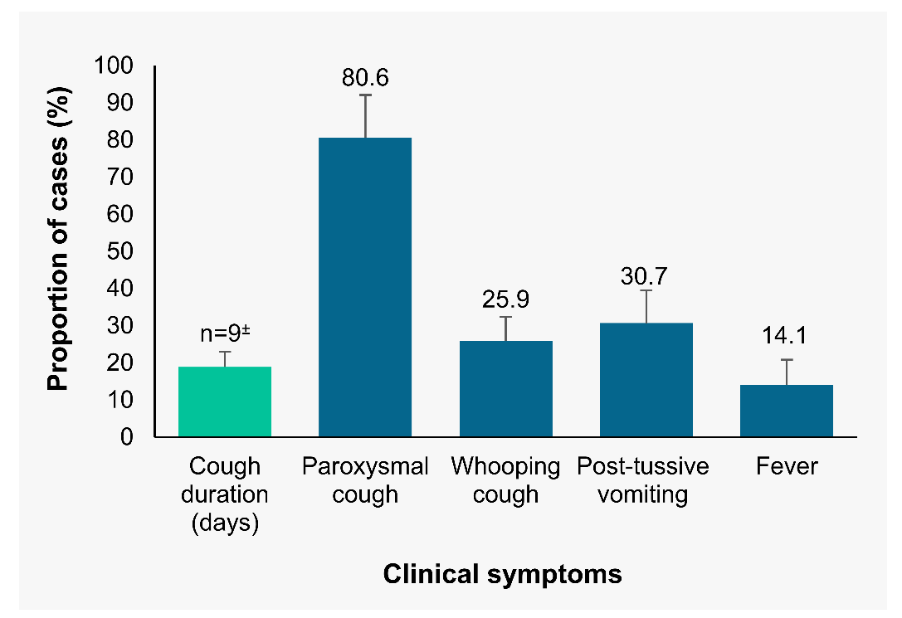


^±^average reported cough duration prior to pertussis diagnosis for all studies that reported this outcome

Error bars: standard error

## Supplementary Figure 3: Predictive value of cough duration^±^


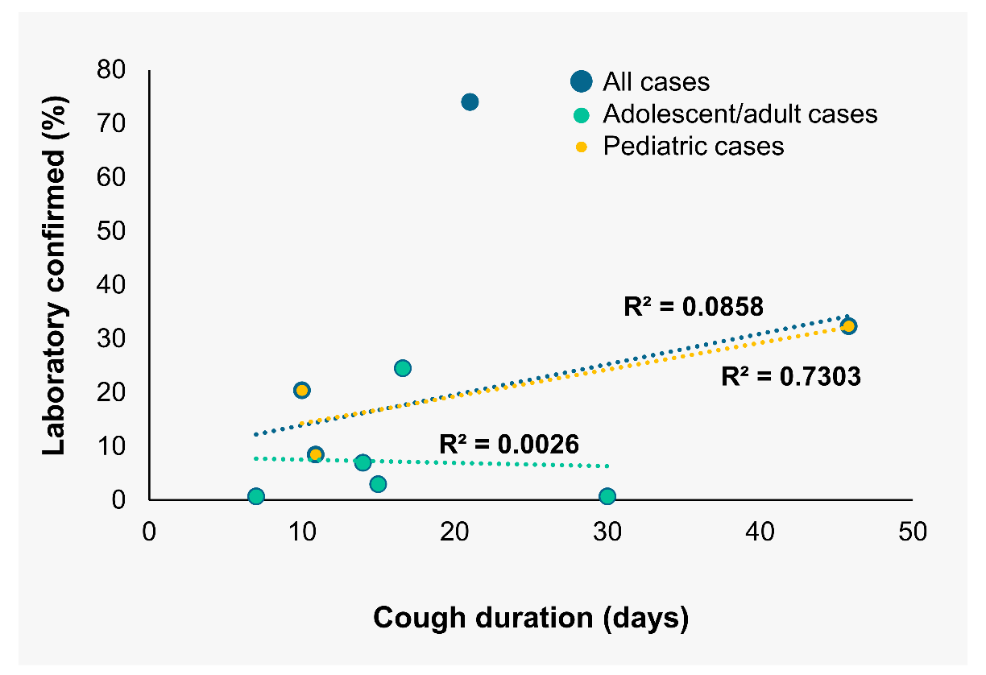


^±^Cough duration before the diagnosis of pertussis

## Supplementary Figure 4: Predictive value of paroxysmal cough^±^

^
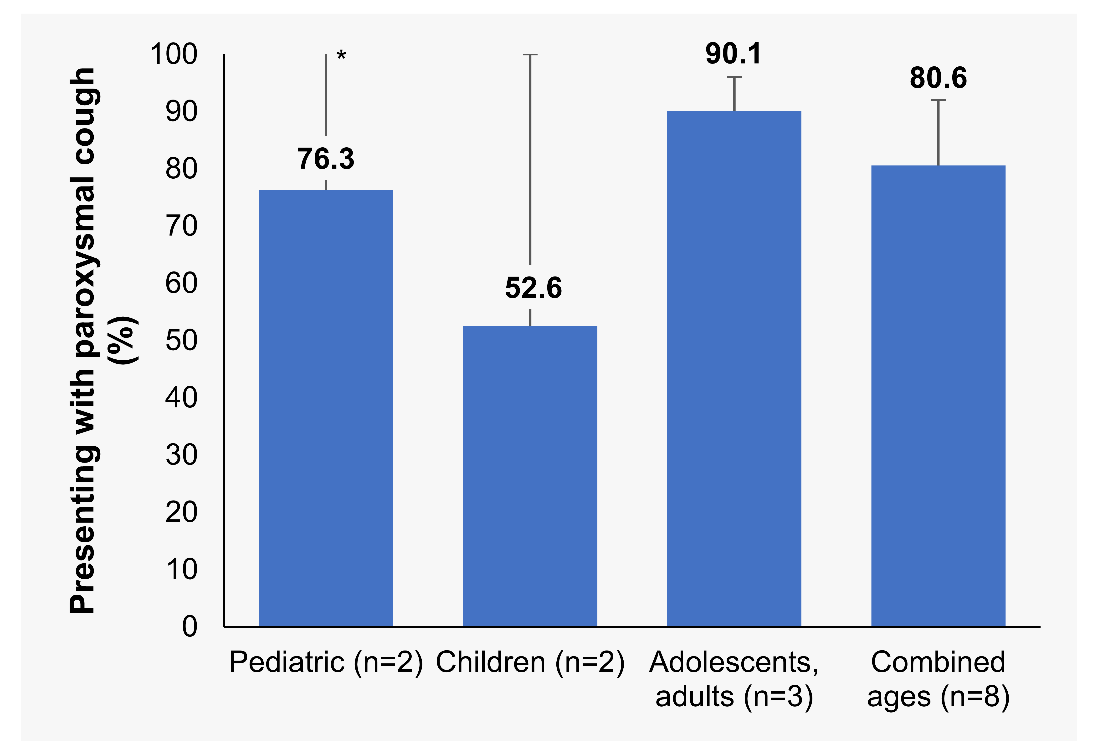
^

Error bars: standard error

^±^Cough before the diagnosis of pertussis

*Standard error: 33.5%

# References

1. Choe YJ, Park YJ, Jung C, Bae GR, Lee DH. National pertussis surveillance in South Korea 1955-2011: epidemiological and clinical trends. Int J Infect Dis 2012; 16:e850-4.

2. Choe Y, Kim J, Park Y, Jung C, Bae G. Burden of Pertussis is Underestimated in South Korea: a Result from an Active Sentinel Surveillence System. Japanese Journal of Infectious Diseases 2014; 67:230-2.

3. Choi WS, Kim SH, Park DW. Seroprevalence of Pertussis in Healthcare Workers without Adult Pertussis Vaccine Use at a University Hospital in Korea. J Korean Med Sci 2018; 33:e321.

4. Han YI, Choi JY, Lee H, Lee T-j. Active Surveillance of Pertussis in Infants Under 6 Months of Age: A Single Center Experience from 2011 to 2013. Korean Journal of Pediatric Infectious Diseases 2014; 21:114.

5. Jang JW, Kang JH, Choi JW, Lee HS, Ma SH. Clinical Characteristics of Pertussis Epidemic in Changwon. Pediatric Infection and Vaccine 2017; 24:37.

6. Kim SH, Lee J, Sung HY, Yu JY, Kim SH, Park MS, et al. Recent trends of antigenic variation in Bordetella pertussis isolates in Korea. J Korean Med Sci 2014; 29:328-33.

7. Kwon HJ, Yum SK, Choi UY, Lee SY, Kim JH, Kang JH. Infant pertussis and household transmission in Korea. J Korean Med Sci 2012; 27:1547-51.

8. Lee KS, Son JS, Chung EH, Bae HK, Lee MJ, Yu J, et al. Prevalence and Clinical Characteristics of Pertussis in Children, Cheonan, Korea. Korean J Pediatr Infect Dis 2009; 16:175-82.

9. Lee SY, Choi UY, Kim JS, Ahn JH, Choi JH, Ma SH, et al. Immunoassay of Pertussis According to Ages. Korean Journal of Pediatric Infectious Diseases 2012; 19:55-60.

10. Lee SY, Han SB, Kang JH, Kim JS. Pertussis Prevalence in Korean Adolescents and Adults with Persistent Cough. Journal of Korean medical science 2015; 30:988-90.

11. Lee SY, Han SB, Bae EY, Kim JH, Kang JH, Park YJ, et al. Pertussis seroprevalence in korean adolescents and adults using anti-pertussis toxin immunoglobulin G. J Korean Med Sci 2014; 29:652-6.

12. Park S, Lee MG, Lee KH, Park YB, Yoo KH, Park JW, et al. A Multicenter Study of Pertussis Infection in Adults with Coughing in Korea: PCR-Based Study. Tuberc Respir Dis (Seoul) 2012; 73:266-72.

13. Park S, Lee SH, Seo KH, Shin KC, Park YB, Lee MG, et al. Epidemiological aspects of pertussis among adults and adolescents in a Korean outpatient setting: a multicenter, PCR-based study. J Korean Med Sci 2014; 29:1232-9.

14. Park S, Oh KC, Kim KS, Song KT, Yoo KH, Shim YS, et al. Role of Atypical Pathogens and the Antibiotic Prescription Pattern in Acute Bronchitis: A Multicenter Study in Korea. J Korean Med Sci 2015; 30:1446-52.

15. Park WB, Park SW, Kim HB, Kim EC, Oh M, Choe KW. Pertussis in adults with persistent cough in South Korea. Eur J Clin Microbiol Infect Dis 2005; 24:156-8.

16. Ryu S, Kim JJ, Chen MY, Jin H, Lee HK, Chun BC. Outbreak investigation of pertussis in an elementary school: a case-control study among vaccinated students. Clin Exp Vaccine Res 2018; 7:70-5.

17. Son S, Thamlikitkul V, Chokephaibulkit K, Perera J, Jayatilleke K, Hsueh PR, et al. Prospective multinational serosurveillance study of Bordetella pertussis infection among 10- to 18-year-old Asian children and adolescents. Clin Microbiol Infect 2019; 25:250 e1- e7.

18. Yoo SA, K.O.; Park, E.H.; Cho, H.S.; Park, C.Y.; Lee, H.R. Epidemiologic and clinical features of pertussis in children. Journal of Korean Pediatric Society 2002:603-8.

19. Yook YS, Jeon JS, Park JO, Kim JK. Laboratory Investigation of Trends in Bacterial Pneumonia in Cheonan, Korea, from January 2008 to September 2017. J Microbiol Biotechnol 2018; 28:1730-5.
